# Navigating Infertility: How Tunisian Men and Women Cope Differently

**DOI:** 10.1192/j.eurpsy.2025.2410

**Published:** 2025-08-26

**Authors:** K. Mahfoudh, S. Hamzaoui, F. Askri, A. Ouertani, U. Ouali, A. Aissa, R. Jomli

**Affiliations:** 1 Department A, Razi Hospital, Manouba, Tunisia

## Abstract

**Introduction:**

Infertility is a significant source of emotional stress for couples worldwide. In Tunisia, it affects approximately 15 to 20% of couples of reproductive age. Studies indicate that women experience higher rates of depression (35% vs. 15%) and anxiety (52% vs. 28%) compared to men, largely due to cultural pressures. For these reasons, understanding these gender differences in coping mechanisms is essential.

**Objectives:**

To analyze the coping styles of infertile couples and identify gender differences in coping mechanisms to inform tailored psychological support.

**Methods:**

We conducted a cross-sectional study involving couples undergoing infertility treatment at a specialized Assisted Reproductive Technology center inTunis. The participants provided information related to socio-demographic data. Coping strategies were assessed using the Brief Cope scale administred in the Tunisian dialect. These strategies were classified into three categories: problem-focused, emotion-focused, and avoidant coping.

**Results:**

A total of 60 infertile couples participated in the study. The average age of men was 41.1±6 years, while the average age of women was 35.07±4 years. Among them, 68% resided in urban areas, and 73% were from a middle socioeconomic background. Educationally, 47% of women held a university degree, compared to 17% of men. Approximately half of the women were unemployed, while 52% of men were employed.

Problem-focused coping emerged as the most frequently utilized strategy (5.93±1.02), followed by emotion-focused coping (5.32±0.82) and avoidant coping (3.95±0.70).

Women significantly employed problem-focused and emotion-focused strategies more than men (p=0.017; p<0.01). They also scored higher in emotional support, expression of feelings, active coping, planning, and religious coping (p<0.05; p=0.01). Conversely, men displayed a greater inclination towards acceptance, distraction, and substance use.

**Conclusions:**

In conclusion, addressing gender-specific coping strategies is essential for providing effective psychological support to infertile couples. Healthcare professionals should promote problem-focused coping to help couples actively manage their challenges.

**Disclosure of Interest:**

None Declared

